# Clinical features of pregnancy-associated aortic dissection and pregnancy outcomes

**DOI:** 10.1186/s12884-026-08716-y

**Published:** 2026-01-31

**Authors:** Mengge Ke, Jingwen Yu, Li Li, Luyao Qian, Guangming Wang

**Affiliations:** 1 Obstetrics, The First Affiliated Hospital of Zhengzhou University, Zhengzhou, 450000 China; 2 Genetic Testing center, The First Affiliated Hospital of Dali University, Dali, 671000 China; 3 Department of Imaging, Dancheng County People’s Hospital, Zhoukou, 477150 China

**Keywords:** Aortic dissection, Pregnancy complications, Cesarean section, Infant mortality rate, Gestational age

## Abstract

**Background:**

Pregnancy complicated by aortic dissection is a rare and severe condition. Due to limited clinical experience, there are currently no standardized management guidelines. Therefore, we summarized and analyzed the clinical data of 33 patients with aortic dissection during pregnancy to gain relevant experience.

**Method:**

A total of 33 pregnant women with aortic dissection admitted to the First Affiliated Hospital of Zhengzhou University and the First Affiliated Hospital of Dali University during 13 years from February 1, 2012 to February 28, 2025 were included. The average age was 32.27 years (range: 23–43 years), and the average gestational age at the occurrence of aortic dissection was 31 ± 7 weeks. There were 17 cases (51.5%) of type A aortic dissection (TAAD) and 16 cases (48.5%) of type B aortic dissection (TBAD). Thirteen cases of TAAD (76.5%, 13/17) and ten cases of TBAD (62.5%, 10/16) occurred during late pregnancy or postpartum. Management strategies were based on anatomical type and gestational age (i.e., prioritizing surgery, medical management, or surgery followed by delivery).

**Results:**

Among the 33 patients, 29 (87.9%) underwent aortic repair surgery, including 14 cases of TAAD (82.4%, 14/17) and 15 cases of TBAD (93.8%, 15/16). A total of 28 patients underwent cesarean section. Among them, 13 cases of TAAD (76.5%, 13/17), 15 cases of TBAD (93.8%). Out of the 33 patients, 6 deaths occurred (18.2%), including 4 TAAD cases with a mortality rate of 23.5% and 2 TBAD cases with a mortality rate of 12.5%. The neonatal mortality rate was 6%, all occurring in TAAD cases.

**Conclusion:**

For pregnant women presenting with thoracolumbar pain and a high suspicion of aortic dissection, timely computed tomography angiography (CTA) examination should be performed to avoid missed or delayed diagnosis. Management of aortic dissection during pregnancy should be based on the anatomical type and gestational age to determine the timing of surgery and delivery, which significantly influences maternal and fetal survival rates.

## Background

Aortic dissection refers to the condition where blood enters the middle layer of the aortic wall through a tear in the inner lining, forming a dissecting hematoma. It is a rare and life-threatening disease [1]. Risk factors for aortic dissection primarily include: structural abnormalities of the aortic wall (such as Marfan syndrome and atherosclerosis), increased aortic wall tension (such as hypertension and aortic coarctation), connective tissue disorders (such as systemic lupus erythematosus), and pregnancy [2].

Pregnancy complicated with aortic dissection is even rarer, with an incidence rate of only 0.0004%. The condition progresses rapidly and is highly dangerous, often leading to the death of the patient. Although rare, pregnancy-related aortic dissection is the third most common cause of maternal mortality due to cardiovascular disease [3]. The Stanford classification is the most commonly used method for categorizing aortic dissections in clinical practice: Dissections involving the ascending aorta are classified as Stanford Type A, while dissections confined to the thoracic descending aorta and its distal segments are classified as Stanford Type B [4]. Aortic dissection during pregnancy most commonly occurs in the ascending aorta, especially Stanford type A, accounting for 79% of all cases [5]. No matter what type of dissection, once ruptured aortic dissection, the condition is dangerous, the probability of successful rescue is extremely low, which seriously threatens the life of the mother and child. Therefore, early identification and whole process management are the key to reduce its mortality.

However, there is currently no global consensus or guideline for the standardized management of aortic dissection during pregnancy. This study retrospectively analyzed the clinical data of 33 patients with aortic dissection during pregnancy, comparing the management strategies and clinical outcomes of different types of dissection. At the same time, it conducted a comparative analysis of the clinical characteristics and pregnancy outcomes between patients who underwent simultaneous cardiac surgery and cesarean section and those who did not receive simultaneous surgery, summarizing their clinical features and management experiences. The objective is to enhance the ability of clinical healthcare providers to recognize and manage this condition early, thereby improving adverse maternal and fetal outcomes.

## Materials and methods

### Patients

These cases were sourced from the First Affiliated Hospital of Zhengzhou University and the First Affiliated Hospital of Dali University. This study was approved by the Ethics Committee of the First Affiliated Hospital of Zhengzhou University (KY-2022-0413) and the Ethics Committee of the First Affiliated Hospital of Dali University (DFY20251015001). The included cases were pregnant women with aortic dissection diagnosed by transthoracic echocardiography (TTE), computed tomography angiography (CTA), magnetic resonance angiography (MRA), surgery, or autopsy during pregnancy or within 12 weeks postpartum [6]. Exclude cases with high clinical suspicion but lacking definitive imaging or pathological evidence; exclude cases with severely incomplete clinical data (such as dissection classification, treatment modality, maternal/fetal survival status) that preclude meaningful analysis; exclude patients with chronic aortic dissection diagnosed prior to pregnancy and undergoing stable follow-up. A total of 37 pregnant women hospitalized for aortic dissection during pregnancy and the puerperium were enrolled from February 1, 2012, to February 28, 2025, across two institutions. After excluding 1 case transferred to another hospital after diagnosis, 2 cases with missing clinical data, and 1 case lost to follow-up after discharge, a total of 33 patients with pregnancy complicated by aortic dissection were included in this study.

### Data collection

Patient general medical history, obstetric history, demographic information, clinical data, echocardiograms, and obstetric and neonatal data were obtained from hospital registration systems and patient records. The following information was collected:

- General data: Maternal age, weight, gestational age at enrollment, family history, general medical history and obstetric history, blood pressure, medication use, echocardiography during pregnancy and delivery, Stanford classification, and pregnancy-related complications such as Marfan syndrome.
- Surgical-related information: Timing and methods of aortic repair, timing and methods of pregnancy termination, temporal relationship between pregnancy termination and aortic surgery, intrapartum circumstances, and postoperative complications.
- Mother-infant Outcomes: survival status, gestational age at birth, birth weight, and neonatal complications.

### Management strategy

**Fig. 1:**
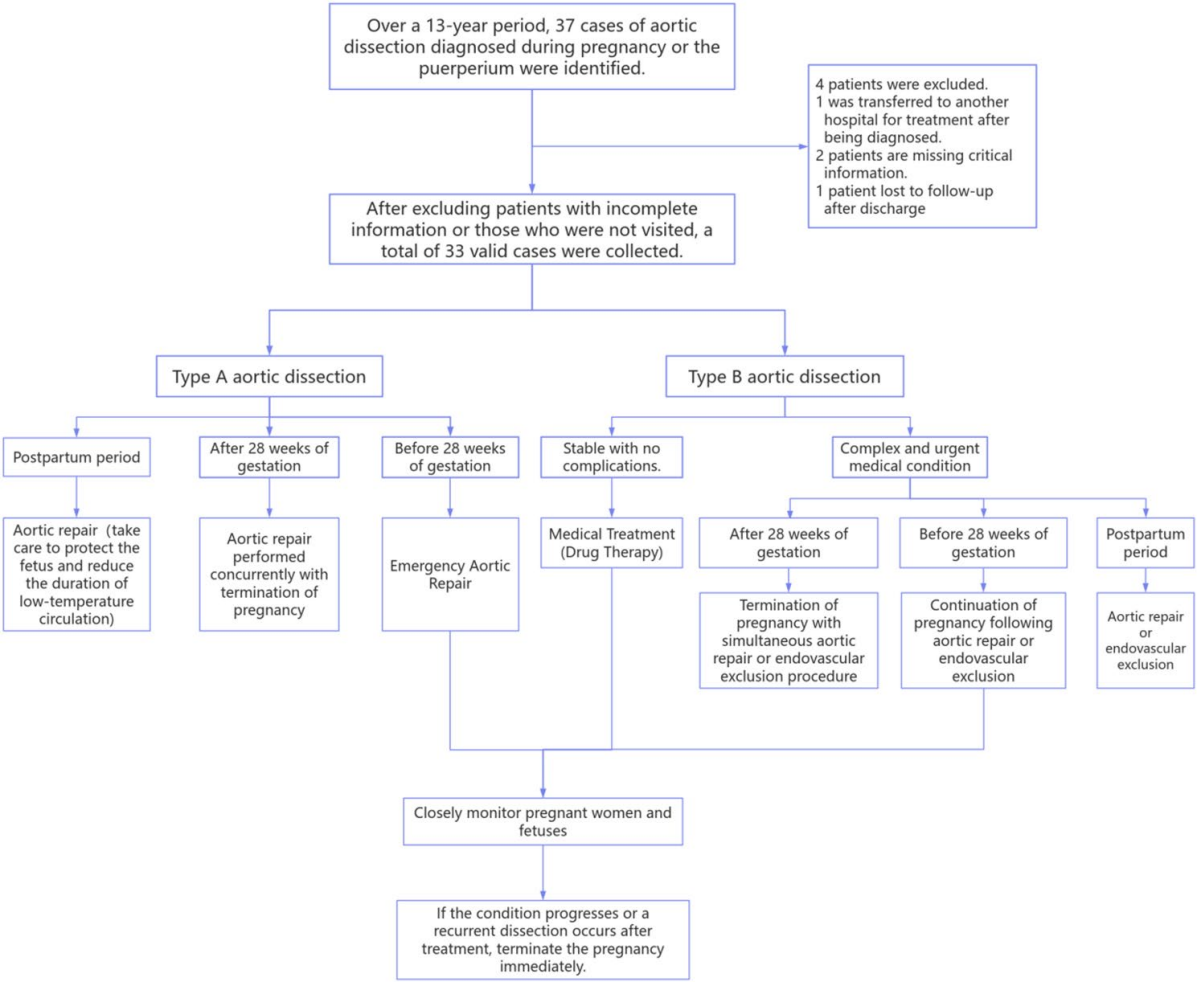
Flowchart of management strategies for aortic dissection during pregnancy and the postpartum period

Classify all cases according to the Stanford classification system. Type A aortic dissection is defined as dissection involving the ascending aorta, while Type B aortic dissection is defined as dissection extending beyond the origin of the left subclavian artery at the aortic inlet. Our management strategy determines the sequence of delivery versus aortic repair, or whether to perform simultaneous surgery, based on the type of aortic dissection and gestational age (Fig. 1). For patients with type A aortic dissection, if gestation is less than 28 weeks, emergency surgical repair or termination of pregnancy will be performed under active fetal monitoring, based on the severity of the dissection and the decision of the patient and her family. When gestation exceeds 28 weeks, an emergency cesarean section will be performed concurrently with aortic repair surgery [7]. For patients with type B aortic dissection, our approach is to deliver the baby first, followed by surgical repair, endovascular repair, or medical management—unless urgent surgical repair is required due to inadequate perfusion, visceral ischemia, persistent pain, or impending aortic rupture [8].

### Patient Follow-up

All survivors (mothers and fetuses or newborns) were conducted primarily through electronic medical record systems, clinic visits, and telephone calls. Attending physicians recorded survival, postoperative complications, reoperations, adverse events, and growth and development of the infant. It is recommended that patients undergo annual CT to assess aortic condition and complications.

### Statistical analysis

We used the Shapiro-Wilk test to find out whether continuous data were normally distributed. While mean ± standard deviation was used for normally distributed continuous variables, median (25–75%) was used for others. Categorical variables were collected as numbers and percentages. Based on the characteristics of our data, Fisher’s exact probability test was used to evaluate the differences in variables between diagnostic groups. Data analysis was conducted using SPSS V.26.0, and a *P* -value < 0.05 was considered statistically significant.

## Result

### Clinical features

The main clinical manifestations and maternal complications are sudden chest pain, shortness of breath, nausea and vomiting. Table 1 summarizes the clinical characteristics of these patients. Among 33 patients with aortic dissection, 17 cases (51.5%) were type A and 16 cases (48.5%) were type B. It is worth noting that only 1 case (3%) occurred during the first trimester, 9 cases (27.3%) during the second trimester, 19 cases (57.6%) during the third trimester, and 4 cases (12.1%) postpartum. This also indicates that the probability of aortic dissection occurring in the middle and late stages is higher. 9 patients developed severe heart failure symptoms, classified according to the New York Heart Association (NYHA) functional classification: there were 7 cases of class Ⅲ (4 cases of type A, 3 cases of type B) and 2 cases of class Ⅳ (1 case of type A, 1 case of type B). Comorbidities: Marfan syndrome in 9 cases (27.3%), hypertension in 17 cases (51.5%), and acute myocardial infarction in 1 case (3%) (Table 1). Patients with Marfan syndrome were predominantly type A (88.9%, 8/9), while only one type B patient had Marfan syndrome (11.1%, 1/9) (*P* = 0.017), whereas patients with concomitant hypertension showed no significant difference between type A and type B dissection (*P* = 0.084). The mean age of these patients was 32.27 ± 5.48 years (type A: 31.53 ± 5.48 years; type B: 33.06 ± 5.56 years), with a mean gestational age of 31 ± 7 weeks (type A: 30 ± 8 weeks; type B: 32 ± 5 weeks).

**Table 1:** Basic clinical characteristics of the patient

Variables	Aortic Dissection				
	Type A	Type B	Total	*P* value	
Patients	17 (51.5%)	16 (48.5%)	33 (100%)		
Age (years), mean ± SD	31.53 ± 5.48	33.06 ± 5.56	32.27 ± 5.48		
Marfan syndrome	8 (47.1%)	1 (6.3%)	9(27.3%)	0.017	
Hypertension	6 (35.3%)	11 (68.8%)	17(51.5%)	0.084	
Positive family history	0(0%)	1 (6.3%)	1(3%)		
Aortic root diameter(mm)	47.35 ± 8.96	34 ± 4.32	40.88 ± 9.74		
NYHA class III ^a^	4 (23.5%)	3 (18.8%)	7 (21.2%)		
NYHA class IV ^b^	1 (5.9%)	1 (6.3%)	2 (6%)		
Gestational age, weeks	30 ± 8	32 ± 5	31 ± 7		
1 st trimester	1 (5.9%)	0 (0%)	1 (3%)		
2nd trimester	3 (17.6%)	6 (37.5%)	9 (27.3%)		
3rd trimester	11 (64.7%)	8 (50%)	19 (57.8%)		
Postpartum	2 (11.8%)	2 (12.5%)	4 (12.1%)		

^a ^Physical activity is markedly restricted. No discomfort occurs at rest, but fatigue, shortness of breath, or angina pectoris may occur with exertion below the level of normal daily activities

^b ^Unable to engage in any physical activity, with symptoms of heart failure or angina persisting even at rest.

### Treatment

Among the 33 patients, 6 deaths occurred (18.2%), including 4 type A cases and 2 type B cases. A total of 29 patients (87.9%) underwent surgical repair, with 13 cases (39.4%) receiving composite graft root replacement (Bentall procedure), primarily in type A patients. Total aortic arch replacement with frozen elephant trunk (Sun’s procedure) was performed in 10 cases (30.3%), all of which were conducted concurrently with the Bentall procedure. Endovascular isolation was performed in 16 cases (48.5%), left subclavian artery embolization in 1 case (3%). Concomitant cardiac procedures included aortic valve repair in 2 patients (6%) and coronary artery bypass grafting (CABG) in 2 patients (6%) (Table 2).

### Type A aortic dissection

**Fig. 2:**
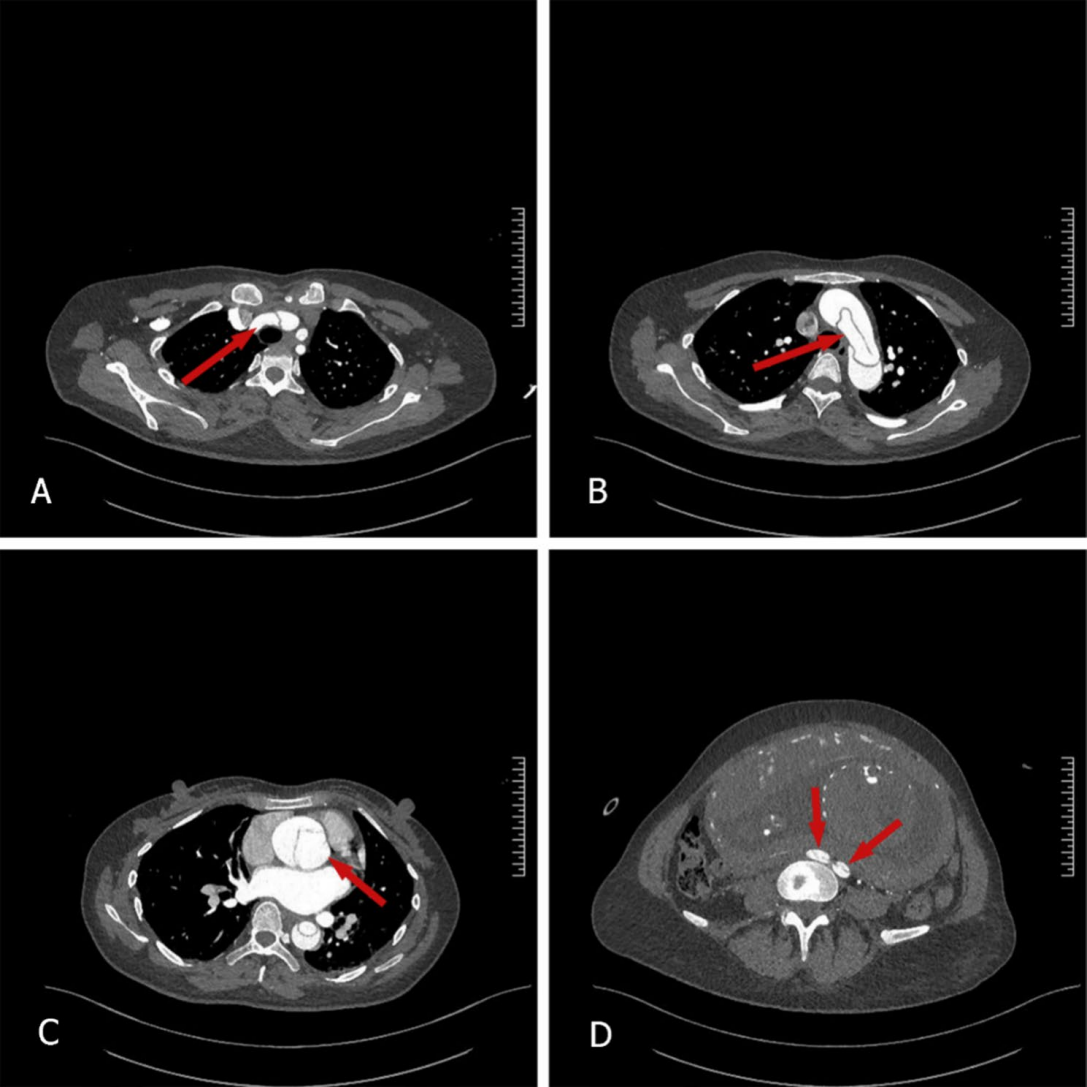
Axial computed tomography images of a 40-year-old woman with acute type A aortic dissection at 31 weeks of gestation, located at the levels of (**A**) the brachiocephalic trunk, (**B**) the aortic arch, (**C**) the ascending aorta, and (**D**) the iliac bifurcation

**Fig. 3:**
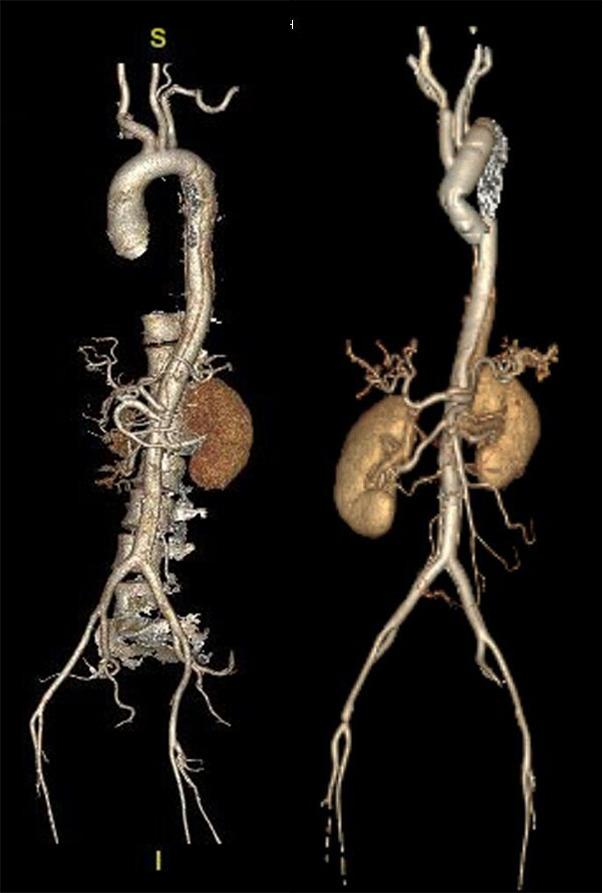
Sagittal reconstruction CT image (left) of a 40-year-old woman with polycystic ovary syndrome presenting with acute type A aortic dissection at 31 weeks gestation. Maternal and fetal survival were achieved via cesarean section, followed by composite root and total arch replacement using the Bentall + Sun’s procedure. CT scan at 2 months postoperatively (right)

Among the 17 patients with type A aortic dissection (Fig. 2), one case was in the first trimester, 3 were in mid-pregnancy, 11 were in late pregnancy, and 2 were in the puerperium. 13 cases (76.5%) underwent cesarean section, with a total of 4 deaths (23.5%), the mortality rate remains relatively high. A case of sudden chest pain one month after amniocentesis-induced termination of pregnancy due to chronic hypertension complicated by severe preeclampsia resulted in death from aortic dissection rupture before surgical intervention could be performed; A 30-week pregnant woman experienced sudden chest pain and underwent an emergency lower segment cesarean section with B-Lynch suturing. She subsequently died from aortic rupture due to aortic dissection before aortic repair could be performed; One case presented with sudden chest pain at 24 weeks gestation accompanied by motor impairment in the left lower limb, the patient died due to ruptured aortic dissection before surgery could be performed. Another case involved a 38-week pregnant woman with acute myocardial infarction. Immediately upon admission, she underwent Bentall procedure combined with Sun’s procedure, coronary artery bypass grafting, cesarean section, and total hysterectomy, one month postoperatively, the patient died from multiple organ failure. 14 cases (82.4%) underwent aortic repair surgery, including Bentall procedures in 12 patients (70.6%), endovascular exclusion in 2 patients (11.8%), and Sun’s Procedure in 9 patients (52.9%). Among these, 2 patients underwent aortic valve repair and 2 underwent coronary artery bypass grafting (Table 2). 10 of the 17 patients (58.8%) underwent cesarean section and aortic repair during the same stage (gestational weeks ranging from 27 to 38 GWs) (Fig. 3), only one patient died among those undergoing concurrent surgery. Patients who underwent surgery during the same period may have a better prognosis.

### Type B aortic dissection

There were 16 patients with type B aortic dissection, including 6 in mid-pregnancy, 8 in late pregnancy, and 2 in the puerperium, among these, 15 cases (93.8%) underwent cesarean section (Table 2). Two deaths occurred (12.5% of Type B cases): A postpartum patient died two days after undergoing endovascular aortic repair due to recurrent aortic dissection rupture; another patient died from aortic dissection rupture 2 h after spontaneous delivery outside the hospital, with no time for surgical intervention. 15 patients (93.8%) underwent aortic repair surgery, including one case (6.3%) who underwent Bentall-Sun’s procedure at 28 weeks (pregnancy terminated via cesarean section at 32 weeks with both mother and infant in good health) and fourteen cases (70.6%) who underwent aortic endovascular exclusion(One case involved a 28-week-pregnant patient without complications who continued pregnancy after conservative management, pregnancy was terminated via cesarean section at 34 weeks, followed by intra-aortic occlusion on the 4th postpartum day, both mother and infant remained healthy. One case involved a patient who underwent endovascular aortic coiling at 26 weeks gestation and continued the pregnancy, with termination via cesarean section at 34 weeks. One case required endovascular aortic coiling combined with left subclavian artery embolization). Among the 16 patients, 5 (31.2%) underwent both cesarean section and aortic repair during the same stage (gestational weeks ranging from 31 to 38 GWs), five pregnant women underwent successful surgeries with favorable prognoses. Neither of the two deceased patients underwent surgery during the same period.

**Table 2:** Data of surgical procedures

Variables	Aortic Dissection			
	Type A	Type B	Total	
Patients	17 (51.5%)	16 (48.5%)	33 (100%)	
Survival	13 (76.5%)	14 (87.5%)	27 (81.8%)	
Death	4 (23.5%)	2 (12.5%)	6 (18.2%)	
Cesarean section	13 (76.5%)	15 (93.8%)	28 (84.8%)	
Natural birth	0 (0%)	1 (6.3%)	1 (3%)	
Management strategies				
Surgical	14 (82.4%)	15 (93.8%)	29 (87.9%)	
Endovascular	2 (11.8%)	14 (87.5%)	16 (48.5%)	
Bentall Procedure^a^	12 (70.6%)	1 (6.3%)	13 (39.4%)	
Sun’s Procedure^b^	9 (52.9%)	1 (6.3%)	10 (30.3%)	
Aortic valve repair	2 (11.8%)	0 (0%)	2 (6%)	
coronary artery bypass grafting	2 (11.8%)	0 (0%)	2 (6%)	
Embolization of the left subclavian artery	0 (0%)	1 (6.3%)	1 (3%)	
Timing of aortic repair and delivery				
Delivery before aortic repair	3 (17.6%)	5 (31.3%)	8 (24.2%)	
Aortic repair was performed at the same time as cesarean section	10 (58.8)	5 (31.3%)	15 (45.5%)	
Aortic repair before delivery	3 (17.6%)	5 (31.3%)	8 (24.2%)	

^a ^Composite Graft Aortic Root Replacement with Coronary Reimplantation

^b ^Tetrafurcated Graft Total Aortic Arch Replacement with Frozen Elephant Trunk

### Fetal and neonatal outcomes

A total of 27 cases (81.8%) of newborns survived delivery(Table 3), 12 cases of type A(70.6%, 12/17), 15 cases of type B(93.8%, 15/16), The live birth rate for Type A aortic dissection is lower than that for Type B. Among them, there were 8 full-term infants (24.2%, 3 with Type A, 5 with Type B) and 19 preterm infants (57.6%, 9 with Type A, 10 with Type B). Two neonatal deaths occurred, both in Type A aortic dissection cases (6%, 2/33). The neonatal mortality rate for type A aortic dissection (11.8%) is significantly higher than that for type B (0%). Both death infants were born at 29 weeks gestation, weighing 1210 g and 1230 g respectively. One was transferred to the NICU and died after unsuccessful resuscitation for pulmonary hemorrhage one day postpartum. The other was taken home by family members and later transferred to a local NICU, where it also died after unsuccessful resuscitation for pulmonary hemorrhage.

**Table 3:** Maternal and infant outcomes

Variables	Aortic Dissection			
	Type A	Type B	Total	
Maternal survival rate	13 (76.5%)	14 (87.5%)	27 (81.8%)	
Maternal mortality rate	4 (23.5%)	2 (12.5%)	6 (18.2%)	
Live birth newborn	12 (70.6%)	15 (93.8%)	27 (81.8%)	
Stillbirth	4 (23.5%)	2 (12.5%)	6 (18.2%)	
Induction of labor or abortion	3 (17.6%)	0 (0%)	3 (9.1%)	
Premature infants	9 (52.9%)	10 (62.5%)	19 (57.6%)	
Term birth	3 (17.6%)	5 (31.3%)	8 (24.2%)	
Neonatal outcomes (Death)	2 (11.8%)	0 (0%)	2 (6%)	
Weight of the newborn(kg)	2.3 ± 0.77	2.8 ± 0.71	2.6 ± 0.76	
Neonatal survival rate	10 (83.3%)	15 (100%)	25 (92.6%)	

Among patients with type A aortic dissection, there were 4 cases of intrauterine fetal death, including 3 cases of induced abortion and miscarriage. One case of induced abortion at 5 weeks of gestation; A Case of Fetal Death at 35 Weeks Gestation with Simultaneous Aortic Surgery and Cesarean Delivery for Fetal Removal; A 27-week pregnant woman underwent amniocentesis-induced termination due to “chronic hypertension complicated by severe preeclampsia and fetal diastolic blood flow reversal.” Among cases of type B aortic dissection, two fetuses experienced intrauterine death. One case underwent endovascular exclusion followed by cesarean delivery to remove the stillborn fetus. The other case experienced intrauterine fetal death two weeks after endovascular embolization and ultimately delivered vaginally.

## Discussion

Aortic dissection during pregnancy is an extremely rare condition. Some patients die before hospital admission, making the true incidence difficult to determine. Previous literature reports an incidence rate of approximately 14.5 per 100,000, with a maternal mortality rate as high as 30% [9]. Among the 33 hospitalized cases we collected, the maternal mortality rate for aortic dissection during pregnancy was 18.2%, with a mortality rate of 23.5% for type A dissection and 12.5% for type B dissection. From this result, it is evident that the mortality rate of type A aortic dissection is significantly higher than that of type B dissection, which also indicates that the presence of type A aortic dissection during pregnancy poses a greater threat to the perinatal period. If pregnant women do not receive systematic treatment, their risk of death increases significantly, with mortality rates rising by 1%–3% per hour. The 24-hour mortality rate reaches 25%, the one-week mortality rate reaches 70%, and the two-week mortality rate reaches 80% [10]. The most critical period in managing patients with aortic dissection begins with timely diagnosis, which requires a high index of clinical suspicion. Only by enhancing awareness of acute aortic dissection and improving the accuracy of initial diagnosis can adverse outcomes be avoided. Unfortunately, the initial misdiagnosis rate for aortic dissection remains high (38%−44%) [11], leading to increased morbidity. In our study, one patient underwent cesarean section due to severe preeclampsia and was diagnosed with aortic dissection one hour after the operation. This case cannot rule out the possibility of delayed diagnosis. Although our dataset lacks direct data on the frequency of misdiagnosis, the clinical courses and complications observed in our cohort are consistent with those reported in other literature. Aortic dissection during pregnancy is highly prone to misdiagnosis as its chest pain symptoms are often similar to those of preeclampsia, labor pain or pulmonary embolism. Previous studies have indicated that delayed or missed diagnoses are key factors leading to adverse maternal and neonatal outcomes [12]. Therefore, a high level of vigilance should always be maintained for this critical condition in pregnant women with atypical symptoms to reduce the incidence and mortality rates. In the existing literature, only approximately 200 cases of aortic dissection during pregnancy have been reported. The rarity and limited experience with this complex condition make clinical diagnosis challenging and preclude the existence of detailed, specific management guidelines [13]. Therefore, we collected data on the etiology, management strategies, and clinical outcomes of 33 such patients over a 13-year period across two hospitals to report our experience.

The risk of vascular events during pregnancy is significantly higher than that in the non-pregnant state. Hormonal changes can affect the basic structure of the aortic wall. The placenta secretes large amounts of estrogen and progesterone that act on the aortic wall, reducing elastic fibers and increasing vascular wall fragility. This renders the aorta susceptible to tearing, leading to the formation of an aortic dissection [14]. During late pregnancy, blood volume increases significantly, and the impact force exerted by blood flow on the aortic wall also increases accordingly, leading to a higher risk of aortic dissection occurring in late pregnancy and the postpartum period. Increased aortic wall tension is more pronounced in women with hypertensive disorders of pregnancy [15], Hypertension was present in 76.6% of patients identified in the International Acute Aortic Dissection Registry [16]. Despite this consensus, our data indicate an exception: there was no significant difference in the prevalence of hypertension between patients with type A and type B aortic dissections, with 51.5% (17/33) of patients presenting with hypertension at the time of onset. In this cohort, 27.3% (9/33) of patients had concomitant Marfan syndrome. Among the 17 patients with type A aortic dissection, 8 were complicated with Marfan syndrome, while among the 16 patients with type B aortic dissection, only 1 was complicated with Marfan syndrome. This result indicates that Marfan syndrome patients are more common in type A aortic dissection, which is consistent with previous studies. A potential mechanism underlying this observation is that women with structural abnormalities in the aortic wall, combined with hemodynamic and vascular changes occurring during pregnancy, face an increased risk of aortic dissection. This risk is particularly elevated in women with a family history of connective tissue disorders and aortic diseases, as well as in individuals with Marfan syndrome [17]. Therefore, for high-risk patients, it is advisable to undergo magnetic resonance imaging (MRI) to assess the entire aorta prior to conception. Patients with aortic root enlargement must undergo preconception evaluation and receive continuous aortic monitoring throughout pregnancy to reduce the risk of aortic dissection during gestation. Generally, patients with aortic root dilation of 4.0 cm to 4.5 cm, those with connective tissue disorders, those with a family history of aortic dissection, or those with indications for surgical aortic valve replacement should plan for surgery prior to pregnancy. Patients with a history of aortic dissection should avoid subsequent pregnancies [18].

All cases of Stanford Type A aortic dissection should undergo prompt surgical intervention to prevent life-threatening complications such as dissection rupture, which can lead to maternal and fetal mortality [19]. In our research group, three cases of gestational type A aortic dissection that did not receive timely surgical treatment died. The 100% mortality rate highlights the urgency and non-negotiability of surgical intervention, providing clear clinical validation for the existing consensus and guidelines advocating immediate surgical repair. The results of this study indicate that for patients with type A aortic dissection, a gestational age of 28 weeks or more can significantly increase the survival rate of the fetus. Compared with single delivery or aortic repair, the strategy of “simultaneous delivery and aortic repair” may lead to better maternal and infant outcomes. However, previous studies have suggested that either delivering first or performing surgery simultaneously can achieve maternal and infant health outcomes [3]. This discrepancy with our findings may stem from the limited sample size, which could explain the inconsistent results. Further research with larger sample sizes is warranted in the future. The primary concern in obstetrics is that extracorporeal circulation during aortic replacement surgery following cesarean section requires substantial anticoagulants, significantly increasing the risk of postpartum hemorrhage. Previous case reports document multiple instances where immediate total hysterectomy was performed after cesarean delivery to prevent major hemorrhage caused by anticoagulants [20, 21]. In this study, 5 cases of type A aortic dissection underwent total hysterectomy during cesarean section, and the patients recovered well after surgery. However, considering the impact of organ resection on female fertility, we have also attempted surgical techniques such as intraoperative uterine packing with gauze strips and B-Lynch suturing, with patients experiencing good postoperative recovery. Similar case reports have been documented in recent years with favorable outcomes, consistent with our findings. This demonstrates that uterine preservation remains safe and reliable when combined with effective hemostatic techniques [22]. For patients undergoing open vascular replacement surgery with plans to continue pregnancy, deep hypothermia has remained our preferred approach for open repair of chronic thoracic aortic dissection and thoracoabdominal aortic dissection in treating this complex pathology [23]. Abrupt termination of cold cycling may lead to fetal hypoxia and potential neurological abnormalities [24], however, shorter cycle-off periods contribute to a favorable prognosis for these patients [25]. One patient in this study underwent Bentall + Sun’s procedure with moderate hypothermic cardiopulmonary bypass. After recovery and discharge, the patient continued the pregnancy. A cesarean section was performed at 2 months postoperatively to terminate the pregnancy. Follow-up revealed normal neonatal development with no neurological abnormalities.

The primary surgical approach for treating Stanford Type B aortic dissection currently involves endovascular exclusion. For cases without bleeding or with unobstructed perfusion to major branches, conservative medical management may also be employed [26]. The first-line drug therapy is beta-blockers [27], which can control blood pressure, relieve pain, and slow the progression of aortic dissection. However, they may affect fetal development, leading to fetal bradycardia and growth restriction [28]. Recent studies suggest that early aortic repair within three months of onset is crucial for achieving complete aortic remodeling in patients with type B aortic dissection [29], though consensus has yet to be reached. In this study, 14 patients underwent endovascular exclusion. One patient died from recurrent aortic dissection rupture 2 days after the procedure, while the remaining 13 patients had favorable outcomes. Due to the small number of cases, current treatment experience remains limited to case reports, making it difficult to determine the optimal therapeutic approach.

Recently, Martino et al. conducted a meta-analysis reporting maternal and fetal mortality rates of 23% and 27%, respectively, among pregnant women with aortic dissection during pregnancy and the puerperium. Among these cases, 67% were type A aortic dissections, significantly higher than type B dissections [30]. Our cohort also exhibited a higher percentage of fetal deaths in Type A aortic dissection, primarily because pregnancies in Type A patients typically occur at earlier gestational weeks. When pregnancy is terminated, the gestational age is smaller, making neonatal survival less likely. The intrauterine fetal death rate for Type A is also higher than that for Type B.

Aortic dissection during pregnancy is a rare occurrence. Although this study conducted a multi-faceted analysis of 33 cases of pregnancy complicated by aortic dissection, its retrospective nature, small sample size, and relatively short follow-up period preclude the formulation of definitive recommendations. Further research involving larger patient cohorts over extended periods is warranted, alongside the exploration of more standardized multidisciplinary clinical pathways to further optimize the management of such high-risk pregnant women.

## Conclusion

In the treatment of pregnancy-associated aortic dissection, protecting both the mother and fetus is considered a hallmark of high-quality medical care—not solely pregnancy-centered, but rather an integrated approach aimed at enhancing maternal survival rates and fetal viability. However, preserving both the mother and fetus simultaneously remains a therapeutic challenge. Without timely and appropriate treatment, pregnancy-related aortic dissection carries a high risk of mortality for both the mother and fetus. For pregnant women presenting with chest or back pain and a high suspicion of aortic dissection, CTA should be performed promptly to avoid missed or delayed diagnosis. The optimal management strategy should be based on the dissection’s anatomical type, gestational age, level of medical care available, and an overall assessment of fetal viability.

## Data Availability

The data sets used and/or analyzed in this study are available from the corresponding author upon reasonable request.

## Abbreviations

TTE: Transthoracic echocardiography
MRA: Magnetic resonance angiography
CTA: Computed tomography angiography
NICU: Neonatal intensive care unit
